# Supplementary material for: Identification of Tumor-Suppressive miR-30a-3p Controlled Genes: ANLN as a Therapeutic Target in Breast Cancer
Source: Noncoding RNA. 2024 Nov 30;10(6):60. doi: 10.3390/ncrna10060060 (PMC11677261; doi:10.3390/ncrna10060060)
Supplement: Supplementary file 1 [file ncrna-10-00060-s001.zip › ANLN supplementary file 1103/ANLN supplementary Figure 1103.pdf]

Supplementary Figure 1

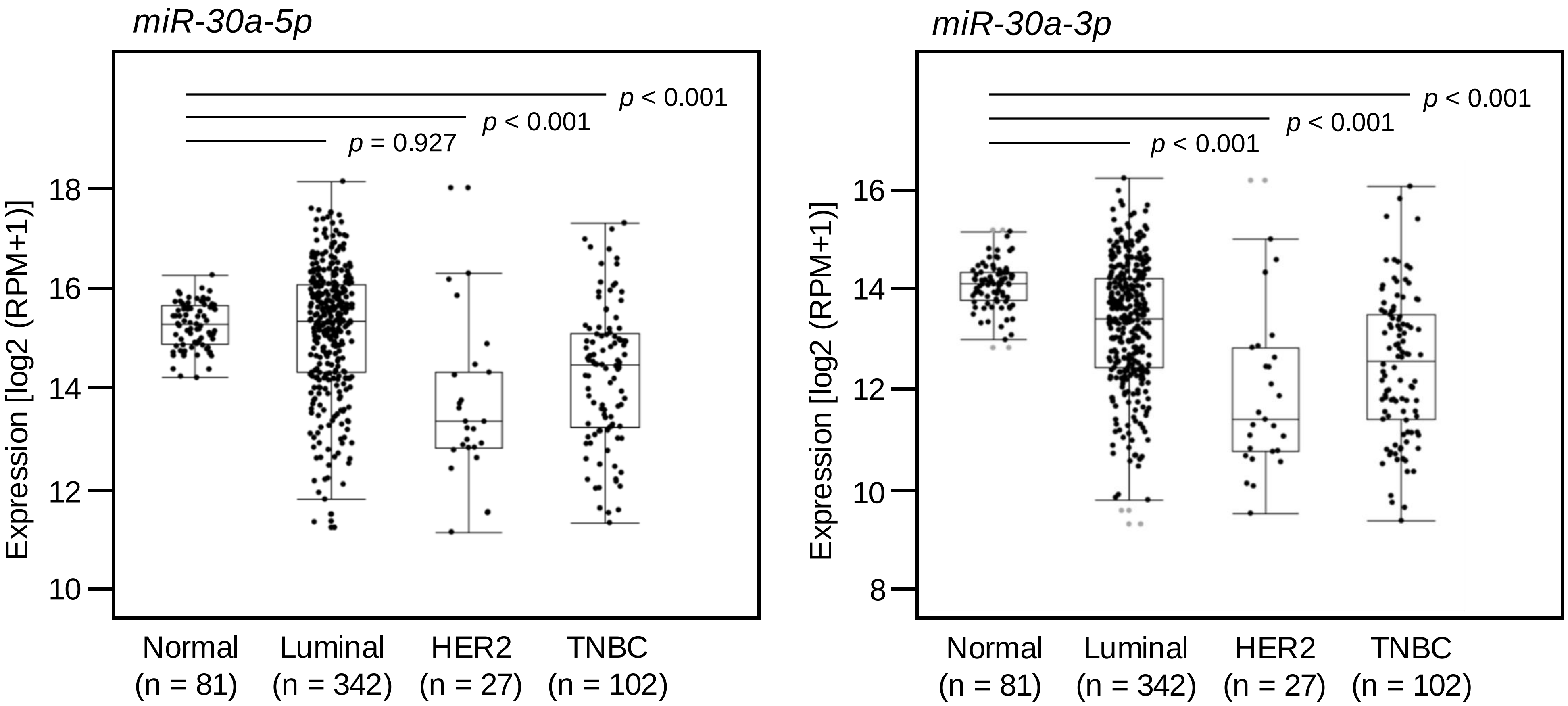

Supplementary Figure 2

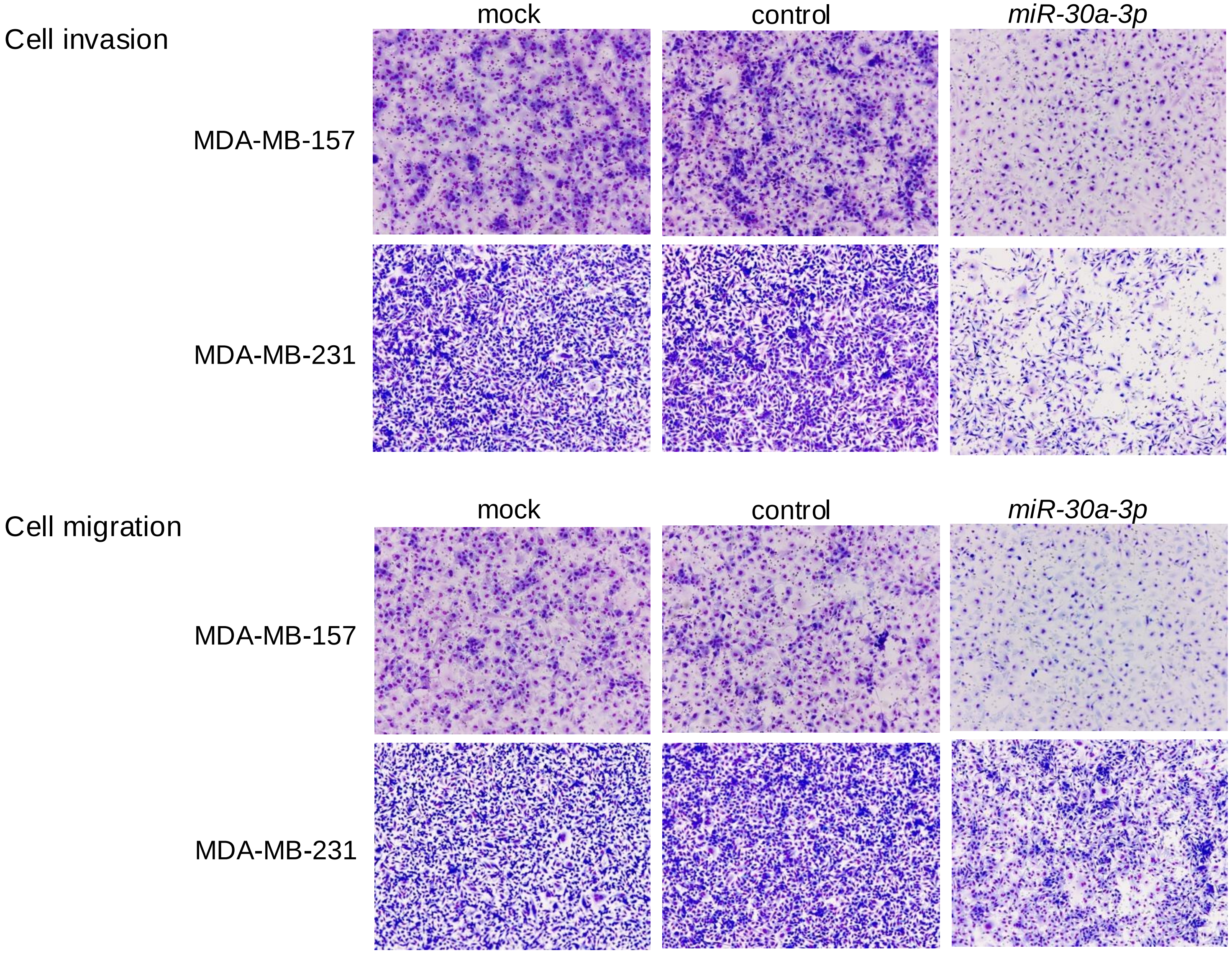

Supplementary Figure 3

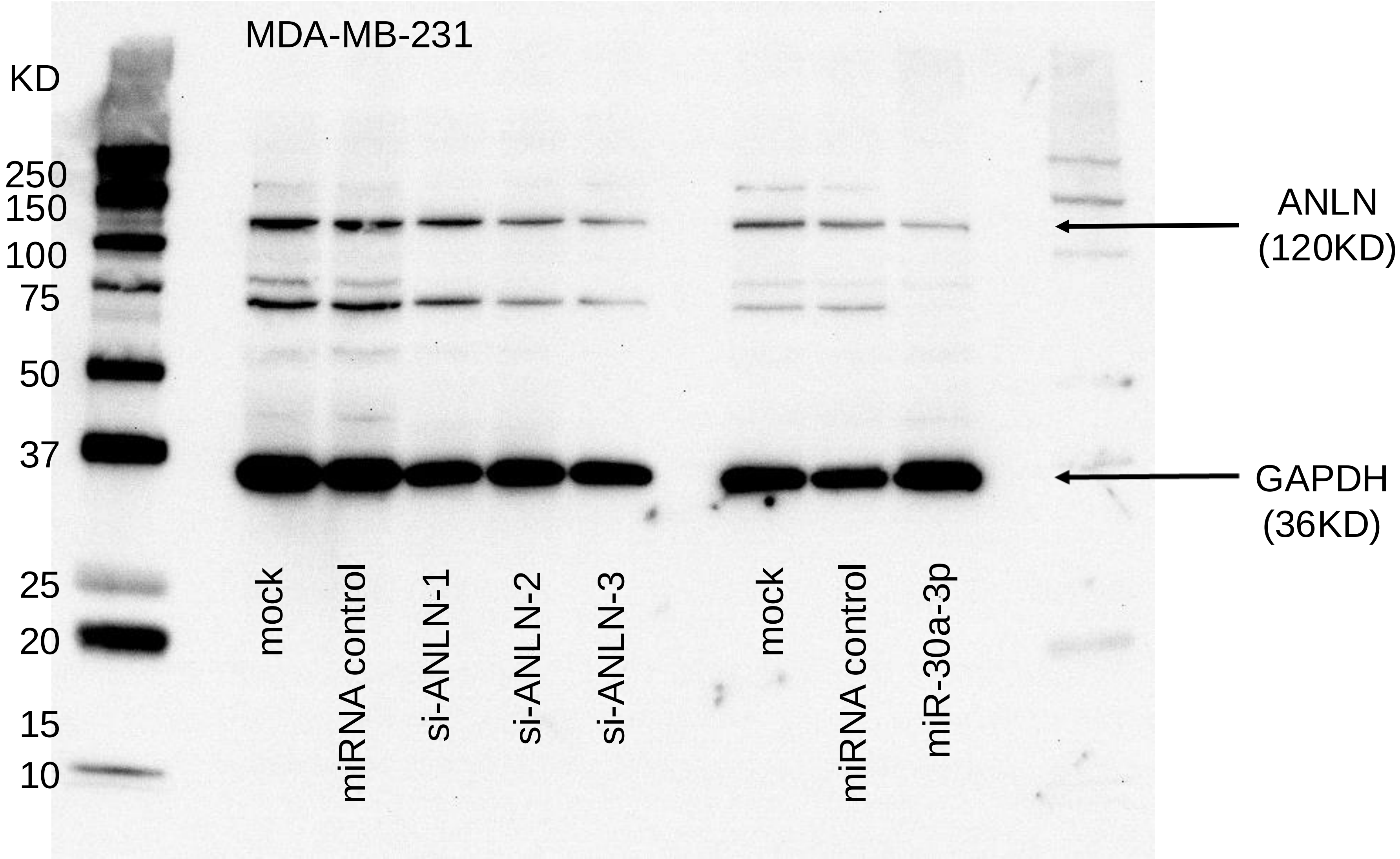

Supplementary Figure 4

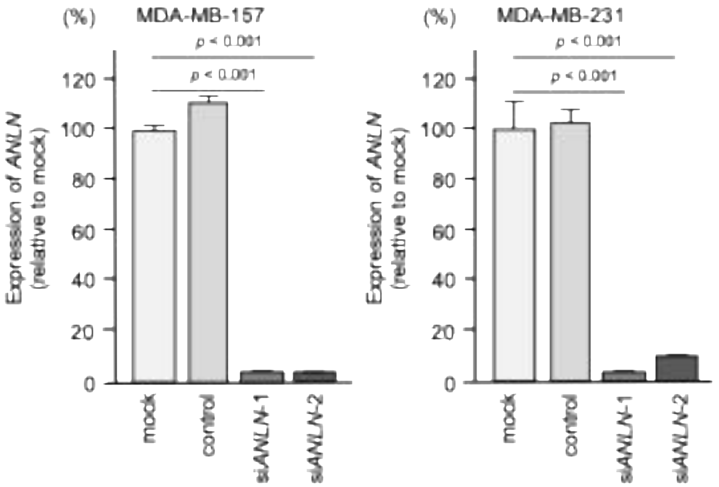

Supplementary Figure 5

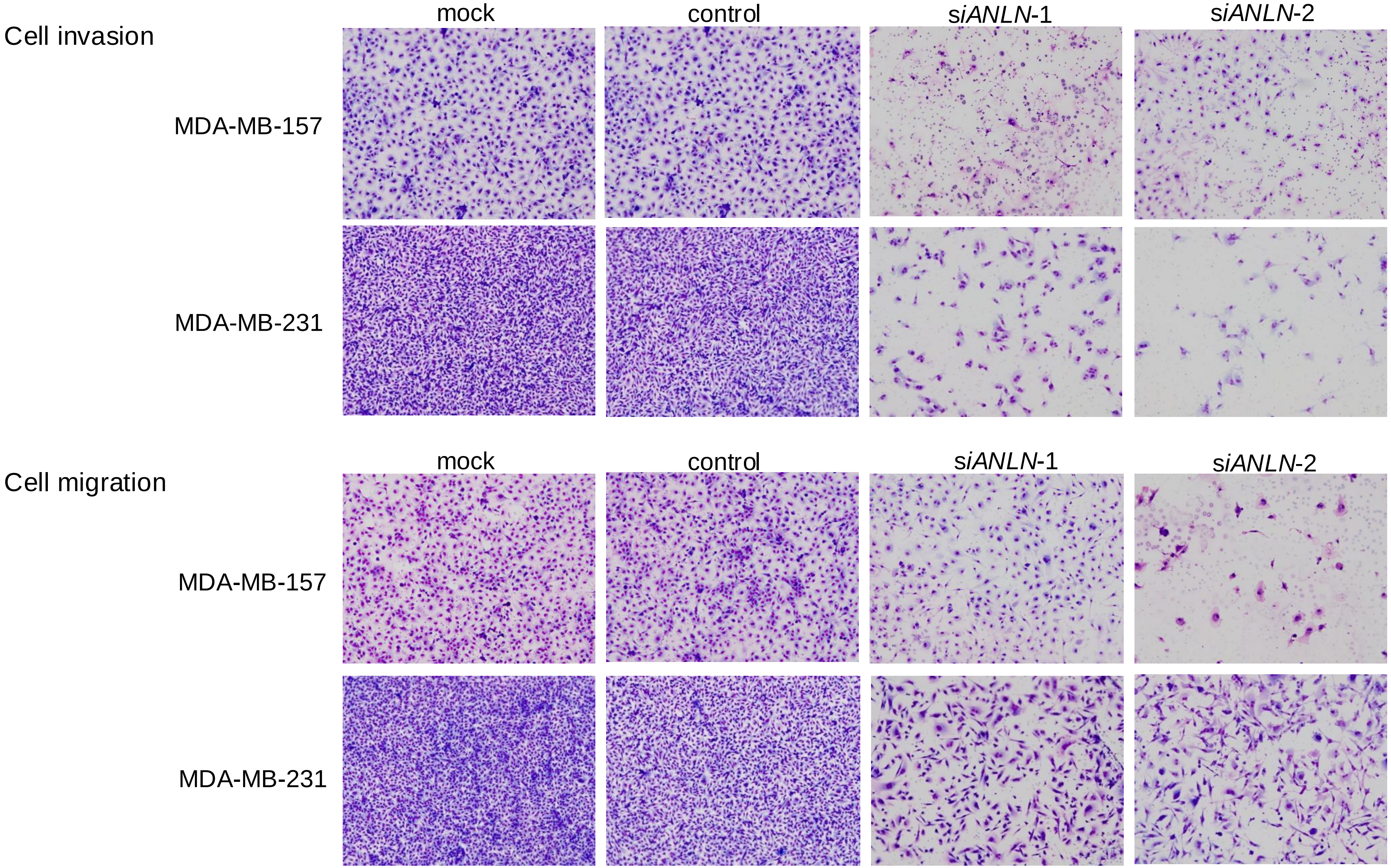

Supplementary Figure 6

actin binding protein (*ANLN*), transcript variant 2, mRNA (NM_001284301)

```
3361 aatcaagttc ttgttgatat tcgcctctgg caacctgatg cttgctacaa acctattgga
3421 aagccttaaa ccgggaaatt tccatgctat ctagaggttt ttgatgtcat cttaagaaac
3481 acacttaaga gcatcagatt tactgattgc attttatgct ttaagtacga aagggtttgt
3541 gccaatattc actacgtatt atgcagtatt tatatctttt gtatgtaaaa ctttaactga
3601 tttctgtcat tcatcaatga gtagaagtaa atacattata gttgattttg ctaaatctta
3661 atttaaaagc ctcattttcc tagaaatcta attattcagt tattcatgac aatatttttt
3721 taaaagtaag aaattctgag ttgtcttctt ggagctgtag gtcttgaagc agcaacgtct
3781 ttcaggggtt ggagacagaa acccattctc caatctcagt agtttttcg aaaggctgtg
3841 atcatttatt gatcgtgata tgacttgtta ctagggtact gaaaaaaatg tctaaggcct
3901 ttacagaaac attttagta atgaggatga gaactttttc aaatagcaaa tatatattgg
3961 cttaaagcat gaggctgtct tcagaaaagt gatgtggaca taggaggcaa tgtgtgagac
```

Vector cloning sequence

```
wild type:
tgatcgtgat atgacttgtt actagggtac tgaaaaaaat gtctaaggcc tttacagaaagttt
deletion type:
tgatcgtgat atgacttgtt actagggt-- -----aaaat gtctaaggcc tttacagaaagttt
```
